# Standardization and implementation of fluorescence molecular endoscopy in the clinic

**DOI:** 10.1117/1.JBO.27.7.074704

**Published:** 2022-02-16

**Authors:** Andrea J. Sterkenburg, Wouter T. R. Hooghiemstra, Iris Schmidt, Vasilis Ntziachristos, Wouter B. Nagengast, Dimitris Gorpas

**Affiliations:** a University of Groningen, University Medical Center Groningen, Department of Gastroenterology and Hepatology, Groningen, The Netherlands; b Technical University of Munich, School of Medicine, Chair of Biological Imaging, Central Institute for Translational Cancer Research (TranslaTUM), Munich, Germany; c Helmholtz Zentrum München (GmbH), Institute of Biological and Medical Imaging, Neuherberg, Germany

**Keywords:** near-infrared, fluorescence molecular endoscopy, standardization, phantoms

## Abstract

**Significance:**

Near-infrared fluorescence molecular endoscopy (NIR-FME) is an innovative technique allowing for *in vivo* visualization of molecular processes in hollow organs. Despite its potential for clinical translation, NIR-FME still faces challenges, for example, the lack of consensus in performing quality control and standardization of procedures and systems. This may hamper the clinical approval of the technology by authorities and its acceptance by endoscopists. Until now, several clinical trials using NIR-FME have been performed. However, most of these trials had different study designs, making comparison difficult.

**Aim:**

We describe the need for standardization in NIR-FME, provide a pathway for setting up a standardized clinical study, and describe future perspectives for NIR-FME.

**Body:**

Standardization is challenging due to many parameters. Invariable parameters refer to the hardware specifications. Variable parameters refer to movement or tissue optical properties. Phantoms can be of aid when defining the influence of these variables or when standardizing a procedure.

**Conclusion:**

There is a need for standardization in NIR-FME and hurdles still need to be overcome before a widespread clinical implementation of NIR-FME can be realized. When these hurdles are overcome, clinical outcomes can be compared and systems can be benchmarked, enabling clinical implementation.

## 1 Introduction



Near-infrared (NIR) fluorescence molecular endoscopy (FME) is an innovative technique that enables *in vivo* visualization of molecular processes in hollow organs by targeting upregulated proteins, overexpressed receptors, or disease-specific biomarkers.^1^^,^^2^ Therefore, it enables real-time highlighting of (early) lesions functioning as a “red-flag” identification method for the endoscopist. Thus, it enables prediction and identification of therapy effectiveness and shows biodistribution of medication. If appropriate targeted fluorophores are used, a highly specific molecular diagnosis can be made without the need for a biopsy.^3^ Potentially, this can improve the detection of precancerous lesions with a flat architecture, which are difficult to detect with only white light endoscopy.^4^ Red-flag identification has been performed successfully in phase I studies of colorectal adenomas and esophageal adenocarcinoma (EAC). In these studies, GE-137 and EMI-137 were used to target C-Met, and Bevacizumab-800CW was used to target VEGFA in all described situations without a therapeutic effect.^5^^–^^10^ Other applications of near-infrared fluorescence molecular endoscopy (NIR-FME) currently under investigation in clinical trials include imaging of drug distribution or the inflammation degree in inflammatory bowel diseases. Moreover, FME can provide simultaneous visualization of white light and fluorescence imaging, enabling the acquisition of both anatomical and biological information. FME has advantages over other well-established and clinically approved optical imaging methods, such as chromoendoscopy,^11^ narrow-band imaging,^12^ and autofluorescence imaging,^13^ because it offers the opportunity to detect specific targets that are unique to the disease. However, FME depends on the existence of dedicated and very sensitive imaging systems, for example, because microdosing of the tracer is used and it operates frequently in the NIR regime, where sensors exhibit reduced sensitivity.^1^^,^^14^^,^^15^

Despite its great potential for clinical translation, FME faces a number of challenges. For example, it is often impossible for the endoscopist to control certain aspects of the data collection, such as the working distance, field of view, or angle of approach. Furthermore, in FME, there is no direct view of the tissue or the possibility for palpation, which limit the possibilities for judgement of the tissue by the operator. These challenges, and more not mentioned, contribute to a lack of consensus in performing quality control and standardization of procedures and systems and complicate discrimination between autofluorescence, cross talk, and genuine fluorescence. If not successfully addressed, these challenges can lead to subjective interpretations by the endoscopist of the visualized data, thereby minimizing possibilities for data quantification and comparison and hampering clinical approval of the technology.^3^^,^^14^^,^^16^^,^^17^

Although these challenges are widely acknowledged, they are still, partly, unresolved. For example, performance standards for manufacturers are known, and phantoms to assess performance metrics are available in other imaging modalities, such as positron emission tomography, x-ray, computed tomography, mammography, an ultrasound imaging. This, however, is not yet the norm for fluorescence imaging.^14^^,^^18^^,^^19^ Contrary to FME, a number of studies for standardization of wide-field surgical fluorescence imaging exist and phantoms are currently being tested for performance assessment of wide-field imaging systems and for the correction and semiquantification of the acquired data. In one such study, Tummers et al.^14^ described several elements that can ensure qualitative and quantitative assessment of the fluorescence imaging performance for each clinical trial. These elements include protocols for imaging during the surgical procedure, specimen mapping and correlation to pathology, and target validation.^14^ Another possibility to address standardization is by developing rigid multiparametric phantoms that enable comparisons between markedly different systems and patients.^18^ Gorpas et al.^16^ recently proposed such a phantom for benchmarking different fluorescence imaging systems. However, translation of such approaches into FME would require significant scaling of the phantoms due to the limited field of view of the employed fiberscopes, i.e., 70 deg, in combination with the working distance that is possible for endoscopy. Moreover, the inherent limitation of the mother–daughter approach (when the fiberscope is guided through the working channel of the endoscope) in FME does not allow for adaptation of the up-to-date proposed wide-field fluorescence imaging standardization methodologies.^14^^,^^20^^,^^21^

The aforementioned challenges are just a few examples that need to be addressed before a widespread clinical implementation of FME can be realized. This paper describes the need for standardization and semiquantification in FME and provides a pathway for setting up a standardized clinical study. Standardization of imaging protocols would improve the reproducibility of research, increase the quality of the data, and ease regulatory approval.^14^^,^^22^

## 2 Standardization

The primary aim of FME is to represent the underlying biodistribution and concentration of a fluorescent probe and, thereby, its biological target. This implies that there should be a relation between the amount of fluorescent probe and signal strength.^22^ To define this signal strength, some form of quantification is necessary. However, (semi-)quantification is difficult due to the numerous variable and invariable parameters that influence the fluorescence intensity, which can be tissue or system dependent and will be described in this section.^14^ By standardizing the majority of the parameters, (semi-)quantification is closer, interpretation becomes less operator dependent, and results are reproducible, which enables a more objective clinical interpretation.

### 2.1 Invariable Parameters

Invariable parameters may refer to the hardware itself, its specifications, and the image quality characteristics of the FME system. These parameters are stable within a measurement or from measurement to measurement.^22^ Each system currently on the market has the same core components, such as a light source for excitation, optical filters, and a detector for sensing the emitted fluorescent signals.^18^ Although the core components are the same between systems, hardware specifications are not, resulting in numerous markedly different systems for clinical use with different sensitivity levels, which is the capability of a camera to detect the fluorescence.^22^

Other invariable parameters influencing sensitivity are (1) dynamic range of the imaging sensor, which is the difference between the smallest and largest signal recorded; (2) spatial resolution of the imaging sensor, which is the number of pixels; and (3) homogeneity and power density of the illumination. Specific for endoscopic systems is the influence of the used fiberscope on the image quality. For example, its resolution (i.e., number of pixels/fibers), its efficiency over time, and the coupling efficiency lead to a different illumination power density with each connection.

### 2.2 Variable Parameters

Variable parameters change during a measurement or from measurement to measurement and can be divided in operating and biological variables. During a procedure, movement is the main factor altering the fluorescence intensity by changing the zoom, focus, distance, and viewing angle from scope to tissue.^3^^,^^22^ In addition, white light is necessary for the endoscopist to guide the probe within the organ, either through the clinical endoscope or through multichannel FME. Although the white light source is usually filtered not to interfere with the detection wavelengths, cross talk is influenced by the relative angle between the fiberscope and the illumination direction. On the other hand, as an endoscopic modality, FME is not affected by ambient illumination or illumination by other devices.

Apart from operating parameters, the tissue optical properties form an important variable parameter by influencing the contrast and attenuation of the signal. Contrast significantly influences the interpretation of FME data, and it depends on the fluorescence emitted by either the intrinsic tissue fluorophores or the nontargeted fluorescence probes and on the cross-talk between the fluorescence and the excitation and/or white light illumination channels. Similarly, attenuation is defined by absorption and scattering and has a direct effect on the signal-to-noise ratio (SNR) of the acquired data.^14^ Furthermore, diffusion, caused by scattering, makes the identification of disease borders or biological structures problematic since it reduces the resolution. These tissue optical properties may differ not only from patient to patient but also from lesion to surrounding tissue within the same field of view.^22^

Tracers form a third variable parameter. Tracers used in FME often consist of antibodies or peptides with a high binding affinity to pathological overexpressed antigens and are labeled with a fluorophore. Ideally, such a fluorescent probe has a low (photo)toxicity level, a specific target uptake, and rapid clearance from nontarget tissue; is stable; and has low cost. These tracers can be given topically or systemically.^2^ Currently, a variety of fluorescent probes are used covering various spectral ranges, from 450 to 1700 nm, showing different biodistributions depending on their ligand, and they may slightly alter in optical properties due to the environment in which the probe is situated. The sensitivity of these probes also depends on the specifications of the imaging system.^22^^,^^23^ Still, partly because of the absence of fluorescence imaging standards and the dependence of the imaging agent and performance of the imaging system, a common approach for regulatory approval is to combine a fluorescent probe with a single system. As such, all outcomes of those trials are not representative for the agent alone but only in combination with the system.^14^^,^^15^^,^^22^ Nevertheless, establishing standardization of FME systems and procedures may facilitate approval of different probes with a specific FME system or the other way around.

## 3 Implementation

### 3.1 Need for Standardization from a Clinical Perspective

From a technological standpoint, standardization is focused on the performance of the imaging system; however, the focus from a clinical standpoint is on the clinical endpoints and interpretation of the fluorescent signal. Therefore, the need for standardization in a clinical environment focusses on the imaging workflow to be able to compare clinical outcomes, validate the fluorescent signal, and correlate the fluorescence to the biology of the patient.

**Fig. 1:**
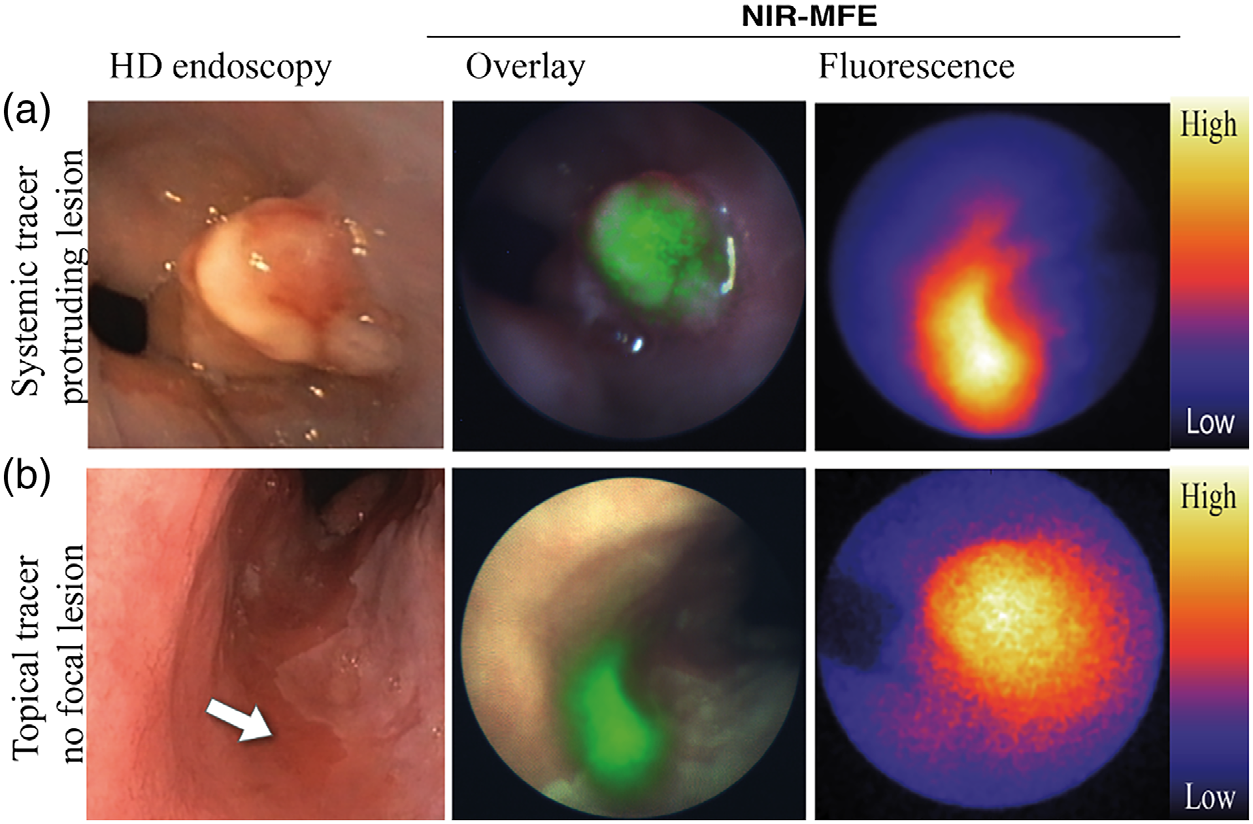
Examples of NIR-FME imaging in the esophagus to identify early EAC, reprinted from Ref. 8. (a) A clearly visible lesion during high definition white light endoscopy (HD-WLE) and also identified by FME. (b) A nonfocal lesion was not identified with HD endoscopy but was identified with FME.

**Fig. 2:**
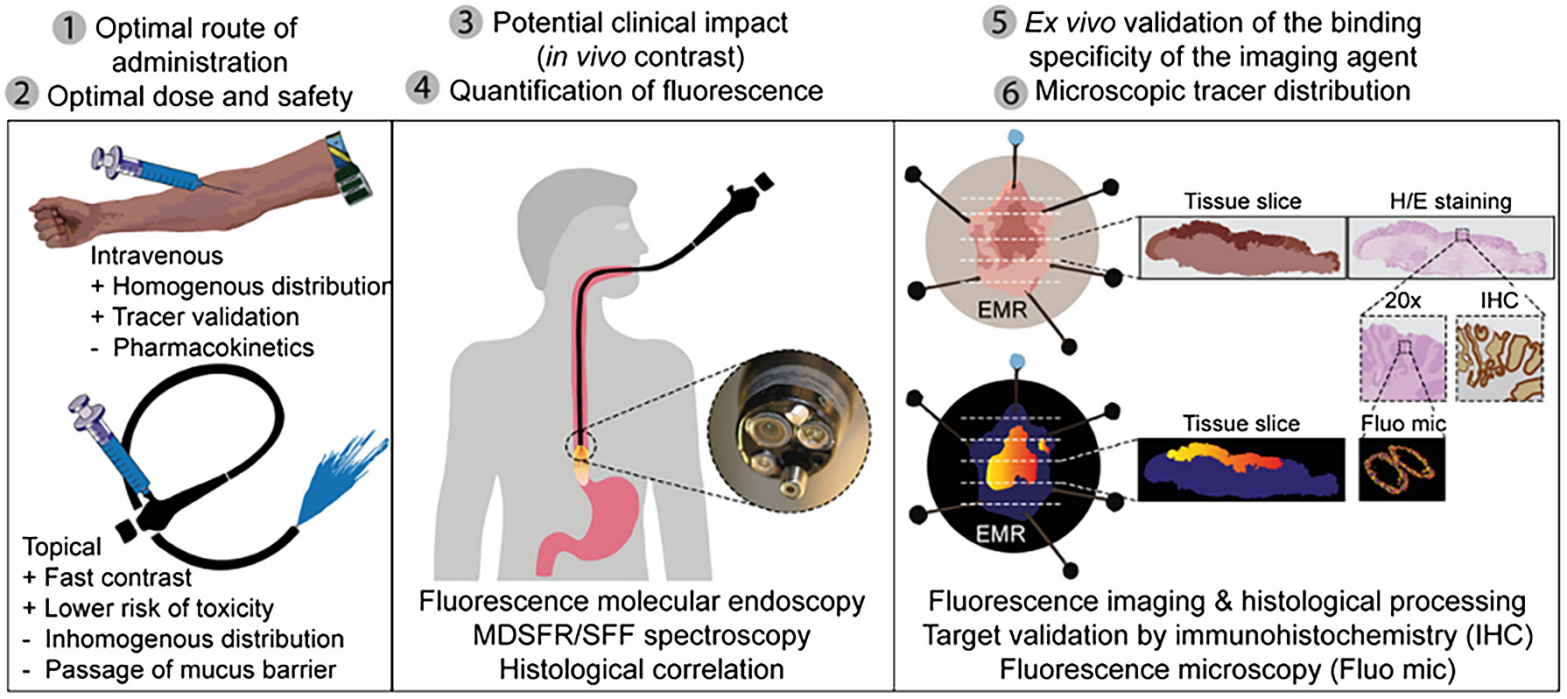
Standardized FME methodology. Six outcomes were defined in the research by de Jongh et al.,^6^ numbered at the top of the figure. Early phase clinical studies should follow this evaluation method to account for all outcome parameters and to evaluate a fluorescent tracer. MDSFR/SFF, multidiameter single fiber reflectance single fiber fluorescence; EMR, endoscopic mucosal resection; H/E, hematoxylin and eosin. Figure reprinted from Ref. 6.

Until now, several clinical trials using NIR-FME have been performed; an example can be seen in Fig. 1 in which Bevacizumab-800CW was used to target dysplastic esophageal lesions.^8^ However, most of these trials had different study designs, which makes it hard to interpret their results or compare them, as acquired study parameters are different and *ex vivo* methods are sometimes used to quantify *in vivo* data. For example, a study done by Burggraaf et al.^5^ using the cMET targeting probe GE-137, describes good visible fluorescence of detected colon polyps in vivo with a target-to-background ratio (TBR), the mean fluorescence signal within the region of interest (ROI) divided by the mean fluorescence signal of the background, of 2.3±1.1 (mean±SD). However, the TBR was measured *ex vivo* in paraffin-embedded biopsies, and no quantification of the intrinsic fluorescent signal was performed.^5^ Therefore, quantitative assessment of probe performance in combination with the system *in vivo* is unclear. On the other hand, a study by de Jongh et al.^6^ used the same cMET targeting probe EMI-137 in patients with Barrett’s esophagus and showed a lower TBR of 1.12 to 1.50 (median range) compared with the previous study. However, the TBRs were acquired *in vivo* with the use of multidiameter single fiber reflectance/single fiber fluorescence (MDSFR/SFF) spectroscopy. MDSFR/SFF spectroscopy corrects the fluorescence signal for the tissue-specific absorption and scattering properties, enabling a quantitative measure of the fluorescence signal.^24^ Thus, the research by de Jong et al. enabled not only acquisition of the *in vivo* fluorescence signal but also the quantification of the *in vivo* signal by calculating the TBR using the intrinsic fluorescence. Although both studies correlated the fluorescence to histopathology, differences in validation of the fluorescent signal in both studies complicates the comparison of TBRs between the two studies. To overcome similar problems and guide validation of a fluorescent tracer in phase I studies, de Jongh et al.^6^ identified several important outcome parameters. Based on these parameters, a standardized FME methodology for clinical studies was suggested (Fig. 2).

When comparing various probes for the same or different indications, similar problems are encountered. For EAC in patients with Barrett’s esophagus, several fluorescent probes have been tested using NIR-FME. However, the absence of standardized validation of the fluorescence signals leads to a difficult comparison between probes.^5^^,^^6^^,^^9^^,^^10^ For example, a study performed by Chen et al. used an innovative technique of multiplexed imaging of Barrett’s neoplasia using two fluorescently labeled peptides.^9^ Although a relation between the *ex vivo* fluorescent signal and the expression of the peptide targets was found using fluorescence microscopy, this study did not enable translation to the *in vivo* fluorescent signal. The same phenomenon is seen in the study by Bird-Lieberman et al.,^25^ who used fluorescent-labeled lectins for the identification of dysplasia in patients with Barrett’s. This study focused mainly on *ex vivo* results and the correlation to histopathology. However, no validation of the intrinsic fluorescent signal was performed.

The aforementioned examples are important landmark studies on their own, done by high level research groups leading in the field of clinical application of FME. However, to make the step to widespread clinical implementation of FME and approval by regulatory agencies such as the Food and Drug Administration and European Medicines Agency, there is a need for reliable comparison and validation of results within the field. Consequently, these examples together demonstrate that standardization is a necessary prerequisite for the clinical translation of the technology.^26^

### 3.2 Phantoms

**Fig. 3:**
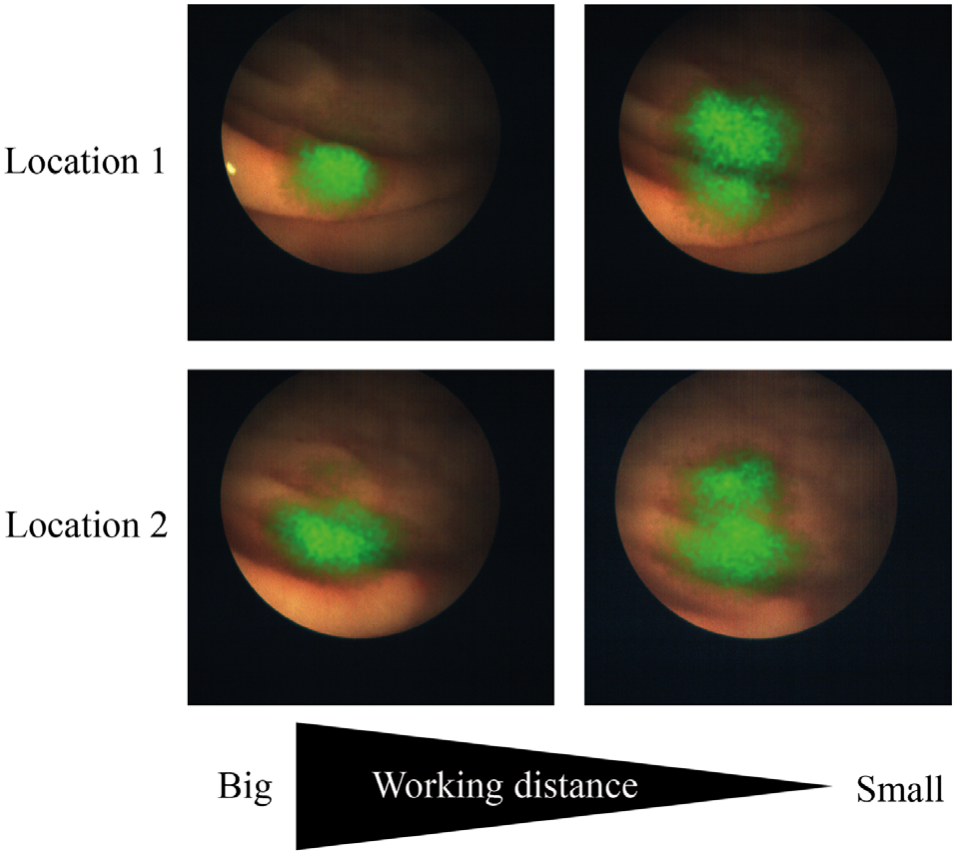
Example of acquired fluorescence signals at different working distances in a polyp identification study. Stills are taken from a video at two different locations as shown in the two rows. A decrease in working distance increases the fluorescence signal, shown in green, and decreases the field of view. Results previously described by Tjalma et al.^27^

Creation of a standardized approach for fluorescence imaging is challenging since many variable and invariable parameters need to be accounted for, which is a complex process. Figure 3 shows an example of the effect of the working distance on the acquired signal in FME. Through standardization based on phantoms or standardized procedures, influences of some of the parameters can be overcome.^18^^,^^28^ Above all, the development of reproducible and reliable phantoms will ensure a continuation of the advancement of innovative optical imaging technologies for improved diagnosis of cancer.^29^ Several research groups designed promising phantoms that pave the way for the establishment of sensitivity limits, performance assessment of cameras, and semiquantification of fluorescent data and thus create standards for FME.^15^^,^^18^^,^^28^

Fluorescent probes are usually present at nanomolar concentrations in the human body. This calls for camera systems with low detection thresholds and, when used to identify small lesions, a high camera sensitivity and a high specificity of the fluorescent probe.^14^ Ever since the development of these imaging systems started, phantoms have been used to test the system and validate physical models, optimize the SNR, perform routine quality control and calibration, and compare performances between systems. In particular, the performance evaluation enables a more uniform system performance between institutions and over time.

**Fig. 4:**
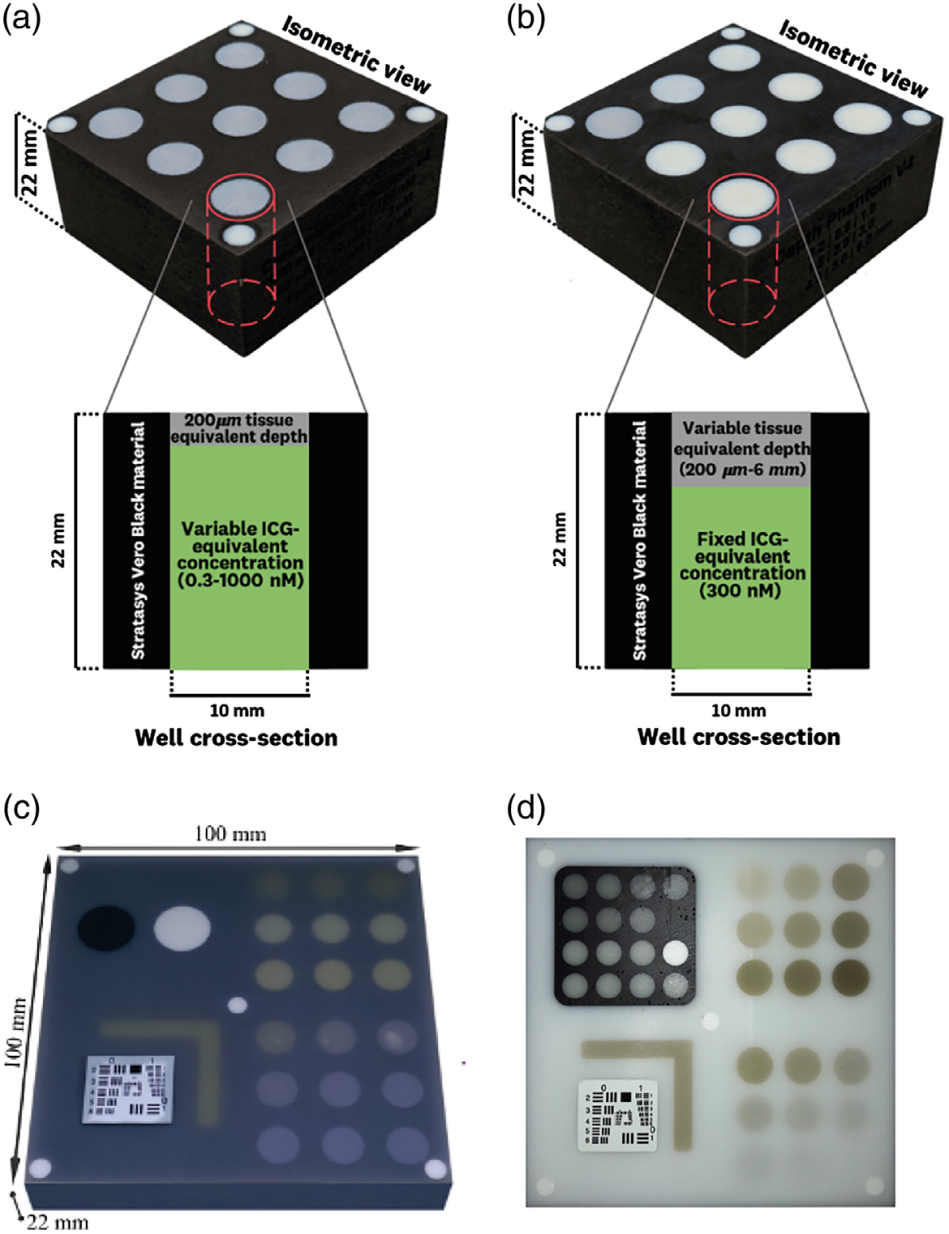
(a), (b) The technical phantom created by Ruiz et al. The vertical cross sections show the buildup of (a) a concentration test and (b) a depth test. Reprinted from Ref. 32. (c), (d) The technical composite phantom created by Gorpas et al. to provide a holistic characterization of a fluorescence system. (c) An earlier version wherein the upper left quadrant provides information about the cross-talk. The upper right quadrant provides information about the sensitivity of the system using different concentrations of a fluorophore and absorption/scattering matrix. The lower right quadrant provides sensitivity information as well based on depth. The lower left quadrant provides information about the resolution, and the five dots in the corners and the middle of the phantom provide information about the field illumination. Reprinted from Gorpas et al.^16^ (d) An improved version of the technical composite phantom created by Gorpas et al.^21^ to provide a holistic characterization of a fluorescence system. The upper left quadrant provides more information on the dynamic range, and the phantom provides the possibility of correcting fluorescence images.

Phantoms can be created either in a technical manner to address specific parameters or to mimic various types of tissue and are referred to as anthropomorphic phantoms. The technical phantoms can be used to address specific parameters, such as the effects of penetration depth, crosstalk between emission and excitation channels, spatial resolution, uniformity and linearity, and sensitivity of the system using different concentrations of a known fluorophore.^30^ However, none of the currently available phantoms provide any means of material characterization (e.g., through NIST standard reference materials), which is essential for the determination of traceable working standards according to the International Systems of Units (SI) to quantify the device sensitivity and compare between systems.^31^ Zhu et al.^31^ did a first attempt using QDots to establish a working standard for the radiance and compared two systems using this phantom. In addition, Ruiz et al.^32^ created a technical long-term stable phantom to test the aspects of commercially available fluorescence imaging systems [Figs. 4(a) and 4(b)]. Using a 3D-printed mold, a phantom consisting of nine wells was created. With this phantom, both a sensitivity test based on varying concentrations and a tissue-equivalent-depth sensitivity test based on different well depths were possible. The performance of the phantom was tested using different commercially available systems in either open air or closed box. Benchmarking based on these phantoms was possible and enabled cross-system comparison.^32^ Kanniyappan et al.^30^ applied three types of phantoms using indocyanine green to establish different performance tests for NIR imaging systems. One of them was a 3D-printed multichannel phantom with white material mimicking tissue scattering to evaluate penetration depth. Each channel was situated at a different depth (2 to 16 mm), and the cross talk of the channels was prevented by adding walls of highly absorbing black material. This phantom formed a simple but effective method for evaluating depth penetration for use in a range of imaging systems.^30^ Although, these phantoms allowed for testing a wide range of performance characterizations and for comparison between systems, clinically, it is not only interesting to compare between systems but also to track day-to-day variances within the system and thus enable standardization of imaging procedure. Therefore, Koch et al. advised the use of disc-shaped composite phantoms, addressing several parameters in one single snapshot. These composite phantoms could also play a key role in semiquantification in different systems using reversion methods and postprocessing of the readouts based on independent measurements of optical properties. Real-time reversion is still difficult since fast processing for correction of the readouts is not sufficient yet.^17^^,^^22^ Gorpas et al.^16^ created a composite phantom offering seamless benchmarking of fluorescence cameras concerning numerous parameters [Fig. 4(c)]. Quantum dots at varying concentrations were used to enable fluorescence imaging, and scattering and absorption in the polyutherane-based phantom matrix and the different structures were simulated by ${\mathrm{TiO}}_{2}$ particles (titanium(iv) oxide) and nigrosine or hemin, respectively. With this phantom, the authors proposed a methodology for comparing fluorescence imaging systems. However, parameters such as dynamic range and correction for the excitation light spatial distribution (fluorescence flat-fielding) were not considered during this study.

To account for these parameters, a new version of the phantom was introduced in 2020, along with a methodology to benchmark fluorescence imaging systems [Fig. 4(d)]. A great advantage of this phantom is that it can also be employed for quality control of a system during a clinical trial. The authors anticipate that this new version of the phantom has the potential to bring comparison of markedly different systems, and thus possibly multicenter trials, even closer. When employing this phantom to restore fluorescence images for the spatial distribution of the excitation light, only working distance and tissue optical properties will influence the readouts.^21^ Nevertheless, using phantoms in a multicenter setting comes with its own hurdles. Cerussi et al.^33^ set up a study into calibration of five different optical instruments, with five different operators and five different phantoms. They suggested that proper phantom use is essential for multicenter studies and that the following aspects should be included in such a phantom: (1) the phantoms need to be stable over a given time (for example, the duration of the trial), (2) the phantoms must be simple to use with chemical mixtures that need to be prepared right before each measurement not being an option, and (3) phantoms should represent reasonable approximations to the absorption and scattering properties of the researched tissue. Another way to set up a multicenter trial could by defining the optical properties using spectroscopy. In this way, the tissue spectra across different instruments, platforms, and locations can be compared. Marín et al.^34^ described this and suggested that it is possible to define a strategy on instrument calibration among centers using this information. During the time of the trial, investigators need to use this technique to demonstrate the validity of the measurements. Above all, the authors believe that the biomedical optics community should adopt a consensus in performance standards and facilitate evaluation and comparison of collected data with different instruments and among different centers, also highlighting the importance of standardization and consensus in the field of FME.

The technical phantoms described above were all designed for wide-field fluorescence imaging systems. Therefore, the question remains as to whether this can be translated to an FME system. Gorpas et al. suggested that the use of a phantom will provide comparable results in FME as in wide-field fluorescence imaging and therefore aimed to create a phantom similar to their previous work but that was smaller and more suitable for FME.^21^

The innovations in 3D printing allow for the creation of not only technical phantoms but also realistic anthropomorphic phantoms that can be used for both calibration and evaluation of (innovative) endoscopic system performance and as a training resource for clinicians.^35^ In addition, exchange platforms such as NIH 3D print exchange allow for sharing of 3D models between researchers, facilitating fast application. Anthropomorphic phantoms should be comparable to the target tissue and include the possibility of incorporating different regions with different optical properties. These types of phantoms should provide long-term photostability and have a fixed geometry. A variety of materials could be used for the design of a phantom. For example, a phantom can be based on an aqueous suspension, hydrogel, silicone, polyester, or even dough.^28^ Although most phantoms are currently based on a general design of the organ, Liu et al. showed in a 3D-printed neurovascular phantom that it is also possible to base it on human subject-specific architecture extracted from an MRI.^35^

Matching tissue properties to anthropomorphic phantoms asks for a comprehensive understanding of the physical and biochemical characteristics of tissue that influence the interaction with light. These properties involve the scattering, absorption, and anisotropy coefficients. A phantom will need both a scattering medium and an absorbing medium. Scattering mediums can be, for example, lipids, polymer microspheres, or metal oxide powder, whereas absorbing mediums can be whole blood, hemoglobin, ink, or a molecular dye. To reach reliable phantoms mimicking the chemistry of tissue as much as possible, biologically compatible structures such as gelatin, collagen matrices, and agar are used. These structures allow for easy inclusion of cellular constituents such as blood or fluorescent molecules but have a limited shelflife.^28^^,^^29^ Further optical properties can be flexibly manipulated using absorbers, scatterers, and fluorophores.^22^

**Fig. 5:**
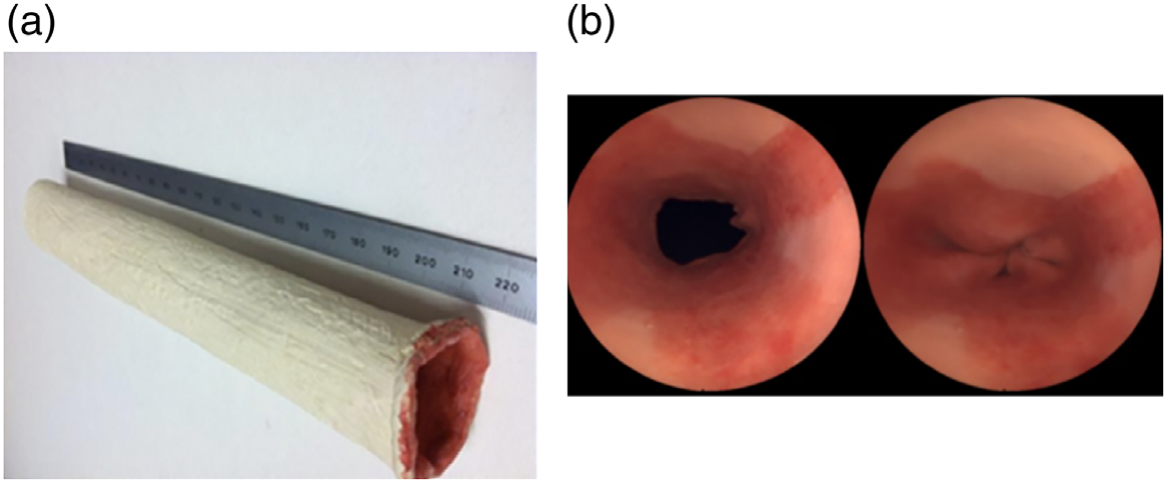
The 3D optical tissue phantom created by Yang et al. to mimic Barrett’s esophagus. (a) The outside the phantom and (b) the inside the phantom, including the closed sphincter. Reprinted from Yang et al.^36^

Yang et al.^36^ described a 3D optical tissue phantom mimicking the human esophagus to validate an FME imaging system (Fig. 5). The synthetic phantom simulated topically labeled fluorescent biomarkers, and surface reflectance was mimicked as accurately as possible based on a Barrett’s esophagus. The phantom was constructed of a paintable elastomeric material flexible enough to mimic the movement in the esophagus and paintable to simulate visual appearance. Autofluorescence and scattering were not incorporated into the phantom mainly because they focused on topical administration of a fluorophore.

**Fig. 6:**
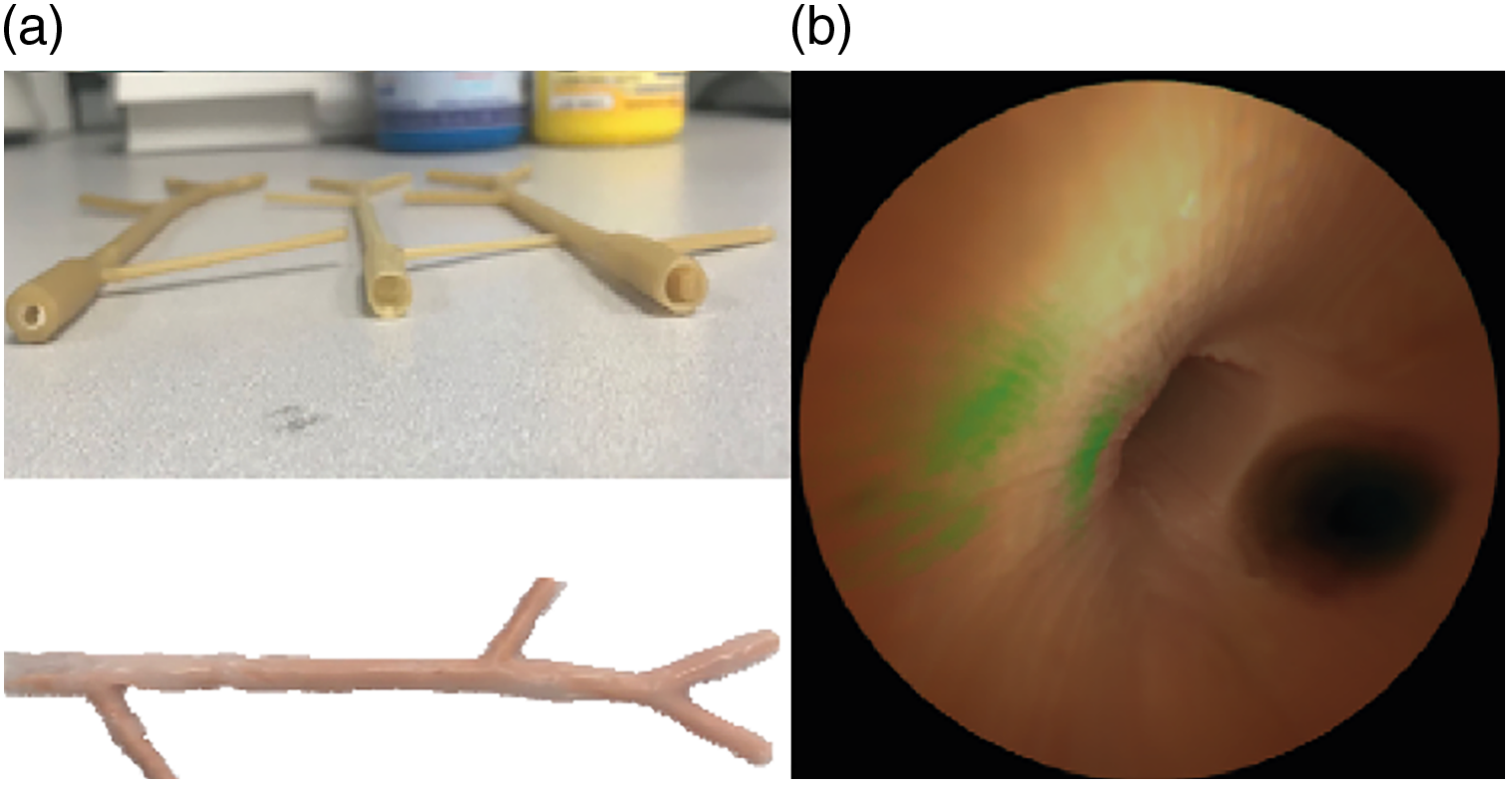
The 3D optical tissue phantom created by Jian et al.^38^ to mimic the gall bladder. (a) Designs and (b) images of the inside, created with fluorescence endoscopy. Adapted and reprinted from Ref. 38.

Anthropomorphic phantoms are not only relevant when validating a system; they can also be of help when practicing. Achterberg et al.^37^ emphasized this when describing the necessities for setting up a clinical trial in fluorescence imaging. Furthermore, Jiang et al.^38^ created a bile duct phantom with NIR fluorescent targets for practicing a clinical study protocol (Fig. 6). This phantom was created from paintable silicone rubber, and the template was created using 3D-printing. Gelatin patches with different concentrations of fluorescent dyes were placed inside the mold. The phantom preparation was repeatable and provided a stable phantom, paving the way for other ductal phantoms.

### 3.3 Interpretation of Data

Absolute *in vivo* quantification is unlikely with the current FME system setup. Nevertheless, in the literature, various methods have been described to semiquantify fluorescence intensities.^14^ Each method resulted in a different quantified fluorescence intensity. Mean fluorescence intensity (MFI) describes the average fluorescence signal in an ROI that may be described in pixels or ${\mathrm{cm}}^{2}$. This intensity is usually shown in arbitrary units (AU). Using AU, however, results in a relative quantification determining differences in fluorescence intensity. By comparing the MFI to a reference background in the same ROI, a relative MFI is constructed. In some other modalities, such as positron emission tomography imaging, usually the maximum value is used; however, the maximum fluorescence intensity is highly susceptible to noise.^14^ Another method is the calculation of the TBR, which can form a measure to address the quality of a fluorescent probe. However, the TBR is highly dependent on the dynamic range of imaging systems, the optical properties of the chosen background, and the specificity of the tracer.^14^ In addition, the SNR or contrast-to-noise ratio (CNR) can be calculated. The SNR is the quotient of the MFI and the standard deviation of the fluorescence intensity outside the measured ROI. The CNR is based on the average fluorescence intensity in the ROI minus the average fluorescence intensity in the background and divided by the standard deviation of the signal in the background.^39^ Although the TBR will show a similar result as the CNR when there is a constant signal, suggesting a clearly visible ROI, the CNR will be more appropriate when there are more fluctuations in the signal and the ROI is not as visible. This makes the CNR applicable to gaining knowledge about the signal fluctuations over time within one patient.^14^ All three of the abovementioned quantification methods (TBR, SNR, and CNR) are dependent on the choice of background as this can significantly affect the results, as shown by Song et al.^40^ Therefore, the minimal TBR threshold is often set subjectively; “high enough for the clinician to see the difference between lesion and background.”^14^ A next step could be to use the International Systems of Units (SI units) to interpret the data. Zhu et al. showed that this was possible using SI units of radiance. Using SI units of radiance might make adoption and implementation of working standards easier and is recommended by the Food and Drug Administration guidelines when performing quality assurance for marketing of a system.^31^

Currently, no standardized procedure of quantification nor definition of the backgrounds exist. What is needed in FME, as a young technology, is consensus. Ideally, the background is uniform and identical everywhere, and the interpretation should be based on a sequence of frames and not one single frame.^40^

## 4 Looking Ahead

This perspective paper describes the challenges in fluorescence imaging and specifically FME. In our opinion, standardization of system performance characteristics and the creation of applicable technical phantoms for FME standardization should be a priority. Similar or equivalent approaches have been proposed by other groups as well,^32^^,^^41^ which highlights the need for consensus within the community, such as was achieved for other molecular imaging technologies.^42^^–^^44^ The first efforts have been made to reach this goal by establishing uniform performance goals for systems and approaches. For example, Pogue et al. wrote a blue paper emphasizing the need for a uniform approach in fluorescence-guided surgery. This blue paper was initiated by the American Association of Physicists in Medicine, who are working toward consensus of the guidelines and standards in fluorescence imaging.^45^ This consensus should be followed by the resizing of phantoms to be applicable for FME standardization and the exploration of (post-)correction of the clinically gathered data. By establishing such a consensus regarding the properties of the used phantoms and (post-)correction in FME, clinical translation of the technology comes closer. Gorpas et al.^21^ already showed this still unique approach when publishing a new version of their wide-field composite phantom [Fig. 4(d)]. When the illumination profile of a system is known, based on a phantom, this can be used to correct the data for effects that degrade the interpretation of the acquired data. However, the correction is only possible when the data are acquired in a stable situation, which cannot be translated yet to a clinical environment.

In addition to applicable phantoms, FME also needs standardization of the clinical procedure itself. Changes in working distance and viewing angle can alter the measured fluorescence as previously described. One way to keep a stable working distance is using anatomical reference points in the white light image^46^ or reference points on the endoscope. However, the preciseness of this approach is questionable. Moreover, it is important to verify *in vivo* results with pathological findings, which is also emphasized in Fig. 2.^6^ De Jongh et al. described this process thoroughly on microscopic and macroscopic levels in a study identifying colorectal polyps with FME.^10^ Pathological slices of 4  μm were both scanned for fluorescence and blinded for fluorescence and inspected by a pathologist. By calculating the MFI in the pathologically selected tumor area, an assessment of the tracer intensity was made. Moreover, as fluorescence can also be influenced by the dosage of the tracer, a standardized dose finding protocol should be followed.^6^^,^^10^^,^^47^ Furthermore, methods such as MDSFR/SFF spectroscopy can provide an objective quantification of the fluorescence signal in these early clinical trials and allow for objective comparison of the results.^6^

If standardization is improved and clinical FME trials can be assessed objectively and compared, a next step would be the introduction of artificial intelligence (AI).^48^ As the level of informational detail increases, the endoscopist is faced with increasingly complex information, resulting in a diagnostic challenge of real-time interpretation. AI methods are already commercially available in endoscopy,^49^ and several computer-aided studies have been performed.^50^ For example, Aihara et al. and Inomata et al. found promising results in a study in which numerical color analysis calculated the green-to-red ratio and found distinguishing values in neoplastic and non-neoplastic tissue during FME.^51^^,^^52^ The commercially available methods used to be focused primarily in the areas of lesion detection and characterization. However, more recently, they are providing assistance in quality measures such as withdrawal times and bowel preparation, both of which can aid in standardization.^49^ For example, Su et al. performed an endoscopy study with 623 patients in which 308 were assigned to automatic quality control concerning polyp detection but also withdrawal and assessment of the bowel preparation.^53^ This last described method of quality control shows the possibilities that AI can provide to standardizing the data collection. In addition to using AI or automated approaches in data assessment, it can also be of use in processing phantom data. Gorpas et al. id this using their composite phantom [Figs. 4(c) and 4(d)]. They created a speeded-up robust feature algorithm to segment different structures of the phantom.^16^^,^^21^ Still, the success of AI implication will dependent on the amount of available and standardized data and the quality of the annotation of the data.^48^^,^^54^ However, more data might be produced using the abovementioned anthropomorphic phantoms.^36^

In summary, the need for standardization in FME and the hurdles that still need to be overcome before a widespread clinical implementation of FME can be realized are described in this paper. The need for standardization to compare clinical outcomes, benchmark imaging systems, and even (semi-)quantify fluorescence signals in FME is high and important to realize clinical implementation. The perspectives provided in this paper provide a pathway to set up a standardized clinical study to improve the reproducibility of research and increase the quality of the data.
